# Neutrophil Extracellular Traps in Cancer Metastasis: From Mechanistic Understanding to Targeted Therapy

**DOI:** 10.3390/curroncol33030156

**Published:** 2026-03-09

**Authors:** Xiaorui Tian, Jintong Na, Xinyi Tan, Fengqiu Dang, Rui Zhu, Liping Zhong, Yongxiang Zhao

**Affiliations:** 1 State Key Laboratory of Targeting Oncology, National Center for International Research of Bio-Targeting Theranostics, Guangxi Key Laboratory of Bio-Targeting Theranostics, Collaborative Innovation Center for Targeting Tumor Diagnosis and Therapy, Guangxi Talent Highland of Major New Drugs Innovation and Development, Targeting Theranostics Research Center of Guangxi Higher Education, Guangxi Medical University, Nanning 530021, China; xiaoruitian@sr.gxmu.edu.cn (X.T.); najt@sr.gxmu.edu.cn (J.N.); tanxinyi@sr.gxmu.edu.cn (X.T.); dangfengqiu@sr.gxmu.edu.cn (F.D.); zhurui@sr.gxmu.edu.cn (R.Z.); 2 Pharmaceutical College, Guangxi Medical University, Nanning 530021, China

**Keywords:** NETs, tumor metastasis, physical barrier, therapeutic target

## Abstract

Metastasis is the leading cause of cancer-related death, underscoring the need to elucidate the key mechanisms behind this process. Neutrophil extracellular traps (NETs) have emerged as critical regulators of tumor progression and metastasis. This review summarizes the primary stimuli and signaling pathways that govern NET formation and outlines the mechanistic roles of NET components in tumor growth and metastatic spread. We focus on environmental and tumor microenvironment-derived factors, including psychological stress, tumor-secreted cytokines, and treatment-related responses, that drive NET formation. The involvement of NETs in multiple stages of the metastatic cascade is discussed, including angiogenesis, tumor cell intravasation and extravasation, circulating tumor cell survival, metastatic colonization, and the reactivation of dormant tumor cells. Additionally, we examine how NETs contribute to the establishment of an immunosuppressive microenvironment. Finally, emerging therapeutic strategies targeting NETs are briefly reviewed, highlighting their potential relevance in metastatic cancer treatment.

## Simple Summary

Metastasis is the leading cause of cancer-related deaths, highlighting the need to understand its mechanisms. Neutrophil extracellular traps (NETs) play a crucial role in tumor progression and metastasis. This review discusses the primary stimuli and signaling pathways driving NET formation, including factors like psychological stress, tumor-secreted cytokines, and treatment responses. NETs contribute to various stages of metastasis, including angiogenesis, tumor cell intravasation and extravasation, circulating tumor cell survival, metastatic colonization, and dormant tumor cell reactivation. They also help establish an immunosuppressive microenvironment. Finally, emerging strategies targeting NETs are explored for their potential in metastatic cancer treatment.

## 1. Introduction

Cancer metastasis is the leading cause of mortality among cancer patients, accounting for over 90% of cancer-related deaths [1]. Unlike primary tumors, which can typically be treated and potentially cured through localized therapies such as surgery and radiotherapy, metastatic cancer represents a systemic disease that affects multiple organs [2]. This occurs through direct colonization of organs, impairing their function, or through modification of the metabolic landscape via altered secretory proteomes, ultimately leading to death. Therefore, the capacity to effectively treat cancer largely hinges on the ability to block or even reverse the metastatic process [3].

The tumor microenvironment (TME) refers to the internal milieu where tumor cells grow and survive, comprising tumor cells, immune cells, endothelial cells, stromal cells, and various extracellular components [4,5]. The TME plays a critical role in the initiation, progression, and metastasis of tumors [6]. Within the TME, chemokines secreted by tumor cells, such as CXCL1 and CXCL8, can recruit neutrophils from the peripheral blood, facilitating their infiltration across the vascular wall into the tumor stroma, where they differentiate into tumor-associated neutrophils (TANs) [7]. TANs exhibit a high degree of functional plasticity, dynamically transitioning between an antitumor phenotype (N1) and a protumor phenotype (N2) and switching between these states [8,9,10,11,12]. The mechanisms underlying this phenotypic transition and the biology mediated by NETs have become focal points of current research [13,14]. Moreover, TANs can synergistically interact with other immune cells, such as tumor-associated macrophages (TAMs) and myeloid-derived suppressor cells (MDSCs), to collectively regulate tumor progression [15,16,17]. NETs are web-like structures released by activated neutrophils, composed of decondensed nuclear DNA or mitochondrial DNA, interwoven with various functional proteins, including histones, neutrophil elastase (NE), myeloperoxidase (MPO), cathepsin G(CG), and matrix metalloproteinase-9 (MMP-9) [18,19].

In 2004, Volker Brinkmann and Arturo Zychlinsky first discovered that NETs serve as a natural immune defense mechanism against pathogen invasion, capable of capturing bacteria, fungi, protozoa, and viruses [20]. Further investigations revealed that NETs could also contribute to ventricular fibrillation [21], mediate atherosclerosis and vascular injury [22,23], and play a role in thrombus formation [24], acute liver injury, and lung damage [25,26]. In recent years, the complex regulatory role of NETs in tumor progression has gradually become a research hotspot [27]. On one hand, NETs capture tumor cells through their DNA framework, and the release of components such as NE, MPO, and antimicrobial peptides directly damages tumor cells, inhibiting their proliferation and metastatic abilities. In addition, NETs can activate CD8^+^ T cells and dendritic cells, enhancing immune responses, while degrading pro-inflammatory factors to limit excessive inflammation and maintain the anti-tumor immune microenvironment. NETs may also inhibit tumor angiogenesis, regulating the blood supply to tumors and further exerting anti-tumor effects [28,29].

On the other hand, contemporary research has increasingly focused on the phenomenon of TANs and NETs accumulating at primary tumors and metastatic sites [30]. NETs can capture circulating tumor cells (CTCs) and form clusters, enhancing their survival in the bloodstream and their capability for distant colonization [31]. The immunosuppressive microenvironment induced by NETs allows tumor cells to evade host immune clearance, thus promoting cancer cell metastasis [32]. Given the growing recognition of the critical regulatory roles of NETs within the TME in tumor development, this review emphasizes the impact of NETosis at various stages of tumor metastasis and its role in establishing the immunosuppressive microenvironment associated with metastatic spread. Furthermore, we systematically summarize the latest research advancements concerning effective therapeutic strategies targeting NETs that promote tumor metastasis.

## 2. Release of NETs

### 2.1. NETosis

Neutrophils, a type of myeloid leukocyte, represent the predominant population of white blood cells in human circulation and act as “first responders” under inflammatory conditions [33,34]. As a crucial component of the innate immune system, neutrophils play multifaceted roles in both inflammation and the tumor microenvironment, with one of their core functions being the formation of NETs through a process termed NETosis [35].

**Figure 1:**
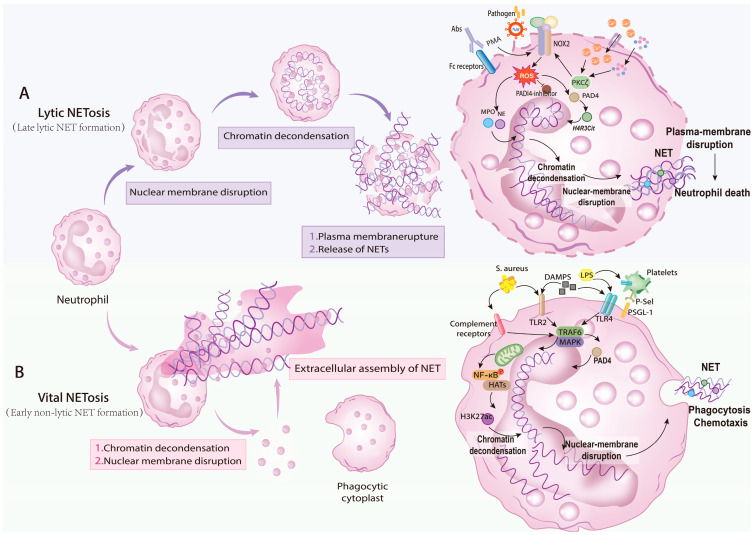
Formation of NETs by Neutrophils through Lytic NETosis and Vital NETosis Mechanisms. (**A**) Lytic NETosis: Major formation steps (**left**) and molecular mechanisms (**right**): Upon stimulation by pathogens, phorbol esters (PMA), interleukin-8 (IL-8), Ca^2+^, inflammatory factors (lipopolysaccharide LPS, tumor necrosis factor TNF), NADPH oxidase 2 (NOX2) is activated to produce ROS, which subsequently activate peptidylarginine deiminase 4 (PAD4), inducing chromatin decondensation. NE and MPO then translocate to the nucleus, facilitating further chromatin unfolding and resulting in nuclear envelope rupture. The decondensed chromatin is released into the cytoplasm, modified by granule and cytoplasmic proteins, eventually leading to the rupture of the plasma membrane and release of NETs, with neutrophil death occurring within hours. (**B**) Vital NETosis: Major formation steps (**left**) and molecular mechanisms (**right**): Staphylococcus aureus can activate signaling through complement receptors and Toll-like receptor 2 (TLR2) ligands, while Escherichia coli can activate Toll-like receptor 4 (TLR4) directly or indirectly through TLR4-mediated platelet signaling, inducing NE translocation to the nucleus within minutes, promoting chromatin unfolding and nuclear membrane disruption. Unlike Lytic NETosis, the modified chromatin is released through a vesicular secretion pathway, and neutrophils remain viable throughout this process. Abs, Antibodies; PKCζ, Protein kinase C zeta; S. aureus, Staphylococcus aureus; DAMPs, Damage-associated molecular patterns; LPS, Lipopolysaccharide; TRAF6, TNF receptor-associated factor 6; MAPK, Mitogen-activated protein kinase; NF-κB, Nuclear factor kappa-light-chain-enhancer of activated B cells; HATs, Histone acetyltransferases; H3K27Ac, Histone H3 lysine 27 acetylation; P-Sel, P-selectin; PSGL-1, P-selectin glycoprotein ligand-1.

Based on whether neutrophils survive or die during the process, NETosis can be classified into two distinct types: Lytic NETosis [36] and Vital NETosis [37] (Figure 1). NETs released following NETosis consist of decondensed DNA forming filamentous extracellular networks. Their core protein composition—including histones, NE, and MPO [38]—collectively acts as a pivotal factor facilitating tumor progression and metastasis [19]. In tumors, TANs can induce NET release through multiple mechanisms, remodeling the tumor microenvironment in a manner that favors tumor cell invasion, proliferation, and metastasis (Figure 1).

### 2.2. Two Distinct Mechanisms of NET Formation

#### 2.2.1. Lytic NETosis

Two prevailing models describe canonical, lytic NETosis. In the model proposed by Grayson and Kaplan, ROS generation via NOX2, translocation of NE and MPO to the nucleus, and subsequent histone modification-driven chromatin decondensation constitute the core events of lytic NETosis [20]. Specifically, pathogen- or stimulus-induced NOX2 activation produces ROS, which activate MPO and drive NE translocation from azurophilic granules into the nucleus. NE proteolytically processes histones and degrades nuclear lamina components such as Lamin A/C, compromising chromatin architecture and nuclear membrane integrity [39]. Moreover, MPO cooperates with NE by binding chromatin and facilitating chromatin decondensation, ultimately leading to nuclear rupture and extracellular release of chromatin bound to NE, MPO, and modified histones [40,41].

An alternative model, advanced by Sørensen and Borregaard, emphasizes PAD4-mediated citrullination of histones as the critical in vivo trigger for NET formation [42]. In this scheme, Ca^2+^ or inflammatory cues activate PKCζ, which phosphorylates and activates the NOX complex to produce ROS. ROS both activate PAD4 and potentiate its activity by inhibiting PAD4 antagonists [43]. PAD4-catalyzed citrullination of arginine residues on histones H3/H4 promotes chromatin relaxation [44]. Together with nuclear lamina degradation, this culminates in nuclear membrane rupture and release of decondensed chromatin complexed with MPO, NE, and citrullinated histones, forming extracellular NETs [45].

#### 2.2.2. Vital NETosis

Vital NETosis is characterized by the release of NETs from neutrophils that remain viable, retaining intact nuclear and plasma membranes as well as a subset of cellular functions [46]. Although PAD4 may participate in its regulation [47], this pathway is typically independent of NOX2-driven ROS production. Rapid Ca^2+^ influx through transporters can activate SK3 channels and trigger mitochondrial ROS (mtROS) generation without requiring NOX2, providing a swift alternative route to NET release [48].

Extrinsic stimuli, such as Staphylococcus aureus, LPS, or high-mobility group box 1 (HMGB1), engage TLR2/4 signaling. TLR2/4 activation recruits TRAF6 and engages MAPK cascades, inducing mitochondrial damage and mtROS production [49,50]. mtROS subsequently activate downstream effectors, including NF-κB, and upregulate histone acetyltransferases (HATs), leading to acetylation at histone H3K27. Chromatin relaxation follows, with relaxed chromatin exported via ESCRT-III-mediated budding or VAMP7-dependent multivesicular-body fusion, preserving plasma membrane integrity [51].

In the context of tumor metastasis, sustained neutrophil activation by tumor-derived cytokines (e.g., IL-8 and G-CSF) or signals from tumor-associated stromal cells can trigger widespread lytic NETosis, releasing large amounts of NETs into the tumor microenvironment and circulation [52,53]. Consequently, lytic NETosis often predominates during inflammatory and metastatic cascades. For example, tumor overexpression of G-CSF expands peripheral neutrophil populations and enhances ROS production, promoting NOX2-dependent lytic NETosis and facilitating pulmonary metastasis [53,54].

### 2.3. Major NET Components and Their Impact on Cancer

NETs comprise multiple antimicrobial factors, among which proteases have been most extensively studied. Proteases bound to NET-associated DNA exert broad cytotoxic effects on target cells. In addition, through proteolytic remodeling of the vasculature and the extracellular matrix (ECM), they promote inflammatory responses and facilitate tumor progression. Among these proteases, NE and CG are particularly important in driving malignancy.

#### 2.3.1. Neutrophil Elastase

NE is a serine protease stored in azurophilic granules and released extracellularly upon degranulation [55]. Within the TME, NE contributes to malignancy through several complementary mechanisms.

First, NET-associated NE can gain access to tumor cell endosomal compartments and, by degrading insulin receptor substrate-1 (IRS-1), drive tumor cell proliferation [56,57]. Second, proteolytic cleavage of the ECM by NE liberates sequestered pro-angiogenic factors such as vascular endothelial growth factor (VEGF) and platelet-derived growth factor (PDGF), facilitating metastatic dissemination [58,59]. However, the notion that NE directly induces epithelial-to-mesenchymal transition (EMT) requires further validation. In ovarian cancer models, NE has been reported to downregulate E-cadherin and activate β-catenin signaling [60], but these observations primarily stem from in vitro studies. It remains unclear whether NE alone—without other TME factors—is sufficient to initiate EMT in vivo. Third, NE induces P-selectin expression on human umbilical vein endothelial cells and promotes vascular dilation, creating a permissive vasculature for tumor cell extravasation [61,62]. Finally, strategies targeting recruited neutrophils and inhibiting NE release confer significant protection in pancreatic cancer models and enhance antitumor immune responses [63].

#### 2.3.2. Cathepsin G

CG is a serine protease encoded within the chymotrypsinogen locus and highly enriched in neutrophil azurophilic granules [64]. Within the tumor microenvironment, CG promotes malignancy through complementary proteolytic and immunomodulatory mechanisms.

Proteolytically, NET-associated CG activates MMPs and downregulates E-cadherin as well as ECM components. These effects collectively enhance tumor cell invasiveness [65]. In a breast-to-bone metastasis model, CG drives a CG-MMP9-TGF-β signaling axis that upregulates MCP-1 and VEGF, stimulates angiogenesis, and accelerates tumor progression [66]. Immunologically, CG is a potent amplifier of inflammation. It chemoattracts macrophages, promotes the production of pro-inflammatory cytokines, and activates IL-1 family members, including IL-18, IL-33, and IL-36, thereby magnifying local inflammatory responses [67,68,69]. Under inflammatory conditions, NE and CG can also degrade the metastasis-suppressor TSP-1, creating a permissive niche for dissemination [70].

Additionally, CG participates in the non-classical renin-angiotensin system by converting angiotensin I to angiotensin II. Nevertheless, while this biochemical activity is established, its functional significance within the TME remains supported only by correlative evidence [71,72].

#### 2.3.3. Matrix Metalloproteinase-9

MMP-9 is a protease released from neutrophils by degranulation and is also associated with NETs [73]. As a pro-angiogenic protease, MMP-9 liberates ECM-sequestered growth factors such as VEGF, thereby stimulating endothelial proliferation, migration, and neovessel formation, which supply tumors with oxygen and nutrients [74].

In addition, MMP-9 degrades collagen and gelatin within the ECM, creating permissive tracks for tumor cell invasion across basement membranes and into the circulation, thereby facilitating metastatic dissemination [69,75]. Acting in concert with other NET components, MMP-9 likely enhances tumor cell invasive capacity during seeding and colonization of distant organs [76].

Nevertheless, although MMP-9 is consistently detected in NET preparations, its specific contribution relative to other NET components remains difficult to isolate. Most studies infer MMP-9 function through correlations or broad-spectrum protease inhibitors; NET-specific MMP-9 ablation experiments have not yet been conducted.

#### 2.3.4. NET-DNA

NET-derived DNA serves both as a structural scaffold and as an active immunomodulatory ligand within the TME. NET-DNA can form physical barriers, engage immunosuppressive pathways, and directly promote metastatic behavior [77].

Mechanistically, NET-DNA acts as a chemotactic and pro-migratory signal. It binds the transmembrane receptor CCDC25 on tumor cells, activating downstream integrin-linked kinase (ILK)-β-parvin-RAC signaling to remodel the cytoskeleton, thereby enhancing directed migration and adhesion [73,78]. In endothelial cells, NET-DNA induces VEGFA expression via an ITGAV/NF-κB pathway, promoting angiogenesis [79].

Pharmacologic blockade of the NET-DNA–CCDC25 interaction (e.g., with mitoxantrone) inhibits NET-driven migration and demonstrates antitumor efficacy across multiple malignancies, including breast and prostate cancer, leukemia, and lymphoma [80,81]. NET-DNA also impairs antitumor immunity: binding to TMCO6 on CD8^+^ T cells suppresses T cell receptor signaling and NF-κB p65 nuclear translocation, weakening cytotoxic responses and accelerating hepatocellular carcinoma progression [82,83].

Nevertheless, although the CCDC25 and TMCO6 pathways have been established, whether NET-DNA binds to additional receptors in specific contexts remains an active area of research. The relative importance of DNA versus DNA-associated proteins in mediating these effects has yet to be systematically elucidated.

#### 2.3.5. MPO-DNA

MPO is a heme enzyme enriched in neutrophils (and present at lower levels in monocytes) that catalyzes the formation of HOCl^−^ from chloride and H_2_O_2_ [84]. MPO is also essential for NET formation: neutrophils from MPO-deficient patients fail to form NETs [85].

MPO-DNA complexes, a structural constituent of NETs, serve clinically as a specific biomarker of systemic NET burden [86]. Elevated circulating MPO-DNA and increased intratumoral citrullinated histone H3 (H3Cit) correlate with higher rates of extra-hepatic metastasis and poorer patient outcomes [65]. Similarly, NET markers are higher in metastatic compared to localized breast cancer patients, supporting a role for NETs in tumor dissemination [87]. In metastatic colorectal cancer, increased intratumoral NETs and raised preoperative serum MPO-DNA levels associate with markedly shorter survival [86].

Nevertheless, although MPO-DNA serves as a useful biomarker, it remains unclear whether MPO itself directly promotes tumor progression beyond its role in NET formation. Further studies using MPO-deficient mice in cancer contexts are needed to distinguish MPO-specific effects from its role in NETosis.

## 3. Role of NETs in Tumor Metastasis

**Figure 2:**
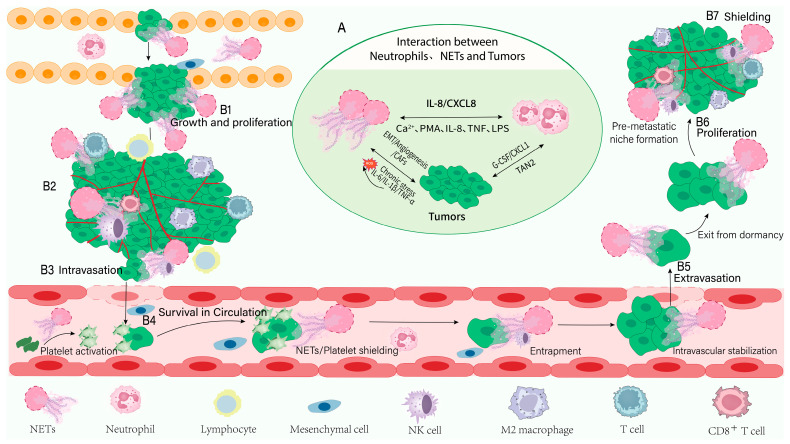
Interactions among neutrophils, NETs and tumor cells, and their contributions to the metastatic cascade. (**A**) Neutrophils release NETs that promote tumor-cell migration and invasion; NETs form web-like scaffolds that capture and shield tumor cells from immune clearance. Neutrophils release NETs that promote tumor-cell migration and invasion, while NETs form web-like scaffolds to shield tumor cells from immune clearance. Tumor-derived factors (IL-8/CXCL8, G-CSF/CXCL1, TAN2) promote NET formation, and NETs in turn facilitate EMT, angiogenesis, CAF activation, and chronic inflammation via pro-inflammatory cytokines (IL-6, IL-1β, TNF-α). (**B1**–**B6**) Steps of NET-mediated promotion of metastasis. (**B1**). Primary-tumor growth—Tumor cells secrete chemokines such as IL-8/CXCL8 that recruit neutrophils and induce NET formation, thereby supporting tumor growth and proliferation. (**B2**). Angiogenesis—NET-associated factors stimulate neovascularization (e.g., via VEGF), supplying the tumor with nutrients and oxygen. (**B3**). Local invasion—NET-bound proteases (NE, MMPs) degrade extracellular matrix components, facilitating tumor infiltration of surrounding tissue. (**B4**). Circulatory survival—NETs interact with platelets to form protective cloaks around CTCs, reducing immune-mediated clearance and increasing CTCs survival. (**B5**). Extravasation and colonization—NETs promote transendothelial migration of tumor cells into distant tissues and support their subsequent engraftment and outgrowth. (**B6**). Dormancy awakening—In pre-metastatic niches, NET-derived signals (for example, CCL5) can reactivate dormant tumor cells and trigger proliferative relapse. (**B7**). Immune suppression—NETs impair anti-tumor immunity through multiple mechanisms, thereby further facilitating metastatic progression. Bidirectional arrows indicate reciprocal induction; unidirectional arrows denote directional promotion of biological processes.

Metastasis is a complex, multistep cascade governed by intrinsic tumor-cell biology and dynamic interactions within the tumor microenvironment [88]. Increasing evidence indicates that NETs, predominantly released by tumor-infiltrating neutrophils, act as critical regulators of cancer progression. Although neutrophils can exhibit context-dependent, and sometimes apparently paradoxical, pro- and anti-tumor activities, NETs themselves have emerged as potent facilitators of tumor growth and dissemination [89]. NETs support tumor-cell survival through multiple mechanisms. Beyond directly stimulating proliferation, they actively promote each stage of the metastatic cascade, including: local invasion; intravasation; protection of circulating tumor cells, and colonization of distant organs [90] (Figure 2).

### 3.1. Stimuli That Promote NET Formation During Metastasis

Increasing evidence indicates that the inflammatory state of the tumor microenvironment links host cells and cancer cells, thereby influencing the metastatic cascade. Neutrophils, the most abundant host inflammatory cells, can affect multiple steps of metastasis [91]. Moreover, within the complex tumor milieu, a variety of factors trigger NETosis through different mechanisms. These stimuli drive the primary tumor into the metastatic process (Table 1).

#### 3.1.1. Non-Tumor-Cell-Derived Factors and Chemotherapy-Induced Mechanisms of NET Formation

Chronic psychological stress and host inflammatory mediators [92,93] promote NETosis by driving ROS accumulation, activating p38 MAPK, and stimulating the cyclin D3/CDK4/6 complex [94,106]. Gut dysbiosis can provoke neutrophil inflammatory activation and Ly6G^+^ granulocyte infiltration, accompanied by marked upregulation of CitH3/MPO^+^ NETs [95]. Activated platelets induce NET formation by releasing HMGB1 or P-selectin; these mediators act both in soluble and cell-bound forms on neutrophils, creating a sustained NETosis-promoting loop [97].

As a major cancer therapy, chemotherapy kills tumor cells but also releases ATP, which can drive tumor cells toward EMT and promote metastasis [98]. Extracellular ATP can additionally activate the NLRP3 inflammasome in surviving tumor cells to induce IL-1β secretion, recruiting neutrophils and stimulating NETosis [99]. Recent studies also showed that while chemotherapy suppresses tumor cell proliferation, it can induce fibroblast senescence and a SASP program (e.g., C3, CXCL1, MIF) that promotes NET formation [113].

#### 3.1.2. Tumor-Cell-Derived Factors That Induce NET Formation

Tumor metabolites and secreted factors enter the TME and, via multiple pathways, influence immune function and angiogenesis to promote malignant phenotypes and metastasis [114]. Tumor-secreted colony-stimulating factor (G-CSF) [100] and the chemokine CXCL1 [115] drive neutrophil production and mobilization into the circulation [82,101,102].

Tumor-secreted S100A7 and TRAPs trigger NETosis via ROS generation and the TLR4/MyD88 pathway, respectively. NQO1 stabilizes and promotes secretion of PPIA, which activates the neutrophil surface pro-invasive protein CD147, stimulating NET formation and NE release [105]. Tumor-secreted fibroblast growth factor 19 (FGF19) activates FGFR4-JAK2-STAT3 signaling in hepatic stellate cells (HSCs), inducing their conversion into iCAFs [106]; iCAFs then secrete complement C5a and IL-1β to activate neutrophils to release NETs, and the resulting DNA–histone–MPO networks remodel the liver metastatic microenvironment [108].

The tumor microenvironment also prolongs neutrophil lifespan and promotes NETosis. HMGB1 released by NETs can activate the TLR9 signaling pathway, further promoting tumor cells to secrete IL-8, thereby forming a positive feedback loop of NET accumulation [111,116,117]. NETs can also activate PI3K/Akt signaling via ROS release, further supporting tumor growth [118]. A KC subset (liver-resident macrophages) secretes DMBT1 that induces a CD62L-expressing KC population; CD62L + KCs activate neutrophils to undergo NETosis via the chemokine CCL8. In addition, binding of DMBT1 to KC membrane MUC1 activates NF-κB signaling in KCs, leading to CCL8 and CD62L expression [107].

### 3.2. The Role Played Throughout the Entire Transfer Process

**Figure 3:**
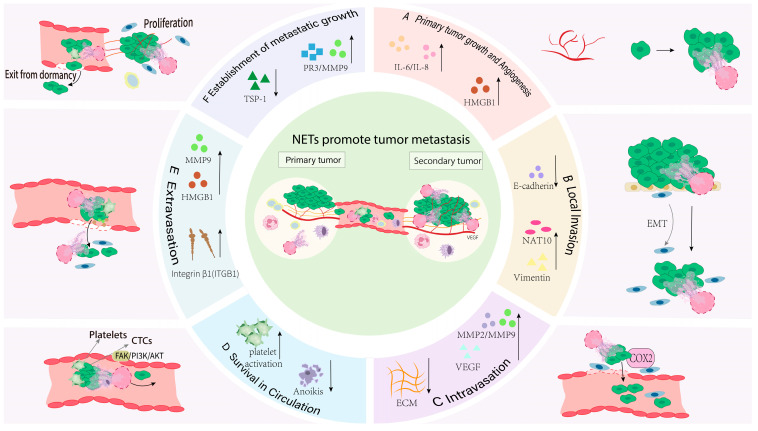
Mechanisms and key factors by which NETs promote the metastatic cascade. (**A**) NETs release pro-tumorigenic mediators (e.g., HMGB1, IL-6, IL-8) that enhance tumor cell proliferation and angiogenesis. (**B**) NETs promote EMT by downregulating E-cadherin and upregulating Vimentin, increasing tumor cell motility and invasiveness. (**C**) NETs facilitate intravasation by promoting degradation of the basement membrane (BM) via MMP2/MMP9 and by releasing VEGF to increase angiogenic and permeabilizing signals. (**D**) NETs enhance survival of CTCs by activating platelets and signaling through FAK/PI3K/AKT, protecting CTCs from Anoikis. (**E**) NET deposits at distant sites secrete HMGB1, histoneases and high levels of TIGB1, increasing microvascular permeability of target organs and promoting tumor cell extravasation. (**F**) NETs stimulate metastatic outgrowth by increasing secretion of PR3 and MMP-9 and by degrading anti-angiogenic/anti-proliferative factors such as TSP-1, thereby supporting proliferation of metastatic. Arrows indicate: Up/down arrows (↑/↓): Protein expression changes that promote the corresponding metastatic step. Solid black arrows: Directionality of biological processes. Gray arrows: Source or direction of action of signaling molecules or cellular interactions.

NETs contribute to tumor progression by promoting primary tumor growth, stimulating angiogenesis, facilitating local invasion, and enhancing intravasation, thereby establishing a foundation for metastasis. NETs exert these effects through multiple core components, including H3Cit, HMGB1, NE, MMPs, and NET-DNA (Figure 3).

#### 3.2.1. NETs Promote Primary Tumor Growth

##### Core NET Components: Histones (Specifically H3Cit) and HMGB1

Inflammation in the tumor microenvironment drives tumorigenesis, and NETs act as key pro-growth mediators. In high-grade glioma, NETs serve as oncogenic markers and directly promote proliferation and invasion [119]. In breast cancer, NET components activate Toll-like receptors, triggering NF-κB signaling and IL-6 production, thereby enhancing tumor cell proliferation [120]. Similarly, in a G-CSF-secreting Lewis lung carcinoma model, NETs formed within necrotic tumor cores support tumor growth [121]. Mechanistically, NETs can reprogram tumor cell metabolism by modulating mitochondrial dynamics and mitophagy, including upregulation of DRP1 and MFN-2, as well as increased PINK1/Parkin expression, which collectively sustain tumor growth [86].

While the proliferative effects of NETs in inflammation-driven tumors are well established, it remains unclear whether they similarly affect tumors with low baseline inflammation, such as prostate cancer. Furthermore, most studies have utilized subcutaneous or orthotopic models, highlighting the need for validation in spontaneous tumor models with a fully intact immune microenvironment.

#### 3.2.2. Angiogenesis

##### Core NET Components: NET-DNA, NE, and MMP-9

Rapid growth of solid tumors generates hypoxia once lesions reach 1–2 mm, and hypoxia subsequently induces angiogenesis to supply nutrients and oxygen [109,122,123]. NETs contribute to this process by stimulating new vessel formation, thereby establishing a malignant feed-forward loop. Mechanistically, NET-DNA activates ITGAV/NF-κB signaling, leading to upregulation of VEGFA and promotion of angiogenesis [124].

In gastric cancer tissues, neutrophil infiltration and deposition of MPO-, CitH3-, and CD66b-positive NETs are associated with endothelial cell activation. NETs induce upregulation of the transmembrane receptor CCDC25 on HUVECs, and NET-DNA binding to CCDC25 activates AKT/mTOR signaling. These events promote endothelial proliferation, migration, and tube formation, enhancing nutrient and oxygen supply to the tumor [78].

Co-implantation of NETs with HCC cells in mice increases tumor volume and CD31 expression, directly demonstrating NET-driven angiogenesis [91]. In a cholangiocarcinoma (CCA) mouse model, PAD4 knockout mice, which are incapable of forming NETs, exhibit markedly reduced tumor progression and angiogenesis, further supporting the central role of the NET-angiogenesis axis in tumor growth and metastasis [79]. Although the pro-angiogenic function of NETs is well supported, most studies rely on exogenous NET administration or transplantation models, highlighting the need for validation in spontaneous tumor models with intact microenvironments.

#### 3.2.3. Local Invasion

##### Core NET Components: NE, MMP-9, and CG

Local invasion refers to the process by which cancer cells detach from the primary tumor mass and invade adjacent normal tissues, a process in which NETs play a critical role [52]. NETs carry proteases, including NE and MMPs, which degrade ECM and basement membrane components. They can also sequester anti-metastatic factors, thereby creating physical pathways that facilitate tumor cell detachment and invasion into surrounding tissues [125].

A more controversial hypothesis is that NETs directly induce epithelial–mesenchymal transition (EMT). Several studies have reported that NET components such as NE, histones, and HMGB1 upregulate mesenchymal markers (vimentin, Snail, Twist) while downregulating E-cadherin in cancer cells [98,101]. However, these findings are primarily based on co-culture experiments or correlative analyses of tumor tissues, and causal evidence—such as demonstrating that NET-specific inhibition can reverse EMT in vivo—is still lacking.

#### 3.2.4. Intravasation

##### Core NET Components: NET-DNA, MMP-2/9, and Histones

Intravasation is the process by which tumor cells traverse the ECM and endothelial basement membrane barriers to enter the blood or lymphatic vessels, becoming circulating tumor cells (CTCs), which is a prerequisite for distant metastasis [126]. This highly coordinated pathological process involves interactions among tumor cells, endothelial cells, immune cells, and soluble factors, with NETs playing a significant role [127,128].

The mechanism can be divided into three stages: Early metastasis: Tumor cells actively remodel the basement membrane and induce angiogenesis, creating physical channels for subsequent invasion into the circulatory system and establishing a nutrient-rich environment [129]. NET-mediated ECM degradation and lymphangiogenesis: NETs promote lymphatic metastasis by inducing lymphangiogenesis and disrupting endothelial barriers [130]. They also stimulate tumor cells to secrete MMP2/MMP9, continuously degrading ECM and vascular basement membranes, thereby clearing the path for intravasation [101]. Enhanced tumor cell motility and vascular remodeling: NETs activate the TLR2/COX-2 axis to induce EMT, enhancing tumor cell motility. Co-localization with the vascular marker CD31 upregulates VEGF and MMP9 expression and remodels tumor vasculature, further increasing intravasation efficiency and metastatic potential [95,131].

Nevertheless, the molecular mechanisms by which NETs specifically enhance endothelial permeability remain unclear. While NET-DNA and histones are known to activate endothelial cells and upregulate adhesion molecules, the underlying pathways—such as VE-cadherin disruption and Rho GTPase activation—have yet to be systematically elucidated.

#### 3.2.5. Survival in the Circulation

##### Core NET Components: DNA–Histone Complexes, MPO, and NE

The pre-metastatic niche (PMN) denotes the supportive microenvironment that primary tumors precondition in distant organs to facilitate the seeding, survival, and outgrowth of arriving CTCs; it represents the “target preparation” stage of metastasis [132]. Within PMNs, NETs recruit neutrophils via the SPP1-CD44-CXCL1 axis, creating NET-dominated microenvironments that preferentially capture CTCs in organs such as the lung and liver [133].

Components of NETs, including DNA, histones, NE, and MPO, activate the coagulation cascade and induce a pro-coagulant endothelial phenotype. This promotes the formation of platelet–NET thrombus complexes, which physically encase CTCs and protect them from immune clearance (e.g., NK cell attacks) and shear stress in blood flow [94,134]. In addition, NET–DNA–histone complexes can directly trap CTCs, forming a sticky network that provides mechanical protection and resistance to anoikis [101]. Both in vitro and in vivo studies demonstrate that NETs are widely present in vascular thrombi within liver cancer tissues and significantly enhance CTC capture [91].

Further studies have shown that NETs induced by cecal ligation and puncture significantly increase CTC capture and liver metastatic burden. This phenomenon is also observed in melanoma models, suggesting that NET-mediated CTC capture is a generalizable process. Therefore, NETs play a central role in PMN formation and subsequent metastasis by promoting CTC retention, immune evasion, and survival in circulation through multiple complementary mechanisms [133].

Although NET-mediated CTC capture is generally considered pro-metastatic, this process may have dual effects. Its ultimate outcome depends on the composition, abundance, and persistence of NETs, as well as the state of the immune microenvironment. NETs may either shield tumor cells or expose them to immune attacks or the cytotoxic effects of NET components themselves.

#### 3.2.6. Extravasation

##### Core NET Components: HMGB1, MMP-9, NE, and NET-DNA

Extravasation is a critical step in the metastatic cascade that determines whether distant colonization succeeds. CTCs must overcome physical endothelial barriers and rely on active cooperation from the distal microenvironment [135]. Proteases released by NETs sustain ECM degradation, weaken the vascular endothelial barrier, and increase local microvascular permeability, thereby promoting tumor cell extravasation [101]. Moreover, NETs deposited in target organs release HMGB1 and histoneases, further increasing microvascular permeability and breaching normally low-permeability tissues [136]. Soluble mediators secreted by the primary tumor precondition the immune microenvironment and cooperate with NETs to amplify these “leakage” effects [137].

NETs can also bridge CTCs to the endothelium via β1-integrins, enhancing adhesion and colonization potential [138]. TGF-β signaling enables NETs to promote extravasation, migration, and EMT in gastric cancer cells [66,139]. Animal studies indicate that NETs present in peripheral blood and ascites facilitate gastric cancer extravasation, proliferation, and metastasis to the liver and peritoneum [140]. Overall, NET deposition in distant organs and the molecules they release increase vascular permeability and colonization efficiency, with CCDC25-mediated CTC adhesion further reinforcing metastatic seeding [141].

#### 3.2.7. Establishment of Metastatic Outgrowth

##### Core NET Components: MMP-9, PR3, NE, and NET-DNA

The lethal consequence of metastasis lies in the ability of tumor cells to colonize and proliferate in distant organs [142]. After escaping immune surveillance, CTCs must survive and form micrometastases [114]. Some disseminated cells enter dormancy for years before resuming proliferation, depending on angiogenesis and support from cancer-associated fibroblasts (CAFs). CAF-secreted β-amyloid can enhance neutrophil ROS production, promote tumor growth, and significantly influence NET assembly [98].

NETs act at multiple points during metastatic outgrowth: (1) Physical trapping and adhesion: The DNA scaffold of NETs provides a matrix that traps CTCs and promotes their adhesion to target endothelium, increasing retention and seeding [125,138]. (2) Protease-mediated ECM remodeling: The NQO1-PPIA-CD147 axis drives NETosis and elastase release, degrading ECM to enhance adhesion and pulmonary seeding [105]. (3) Signaling-mediated NET formation: TGF-β activates a Smad2/3-LIF axis, promoting NET formation. In nude mouse models, interfering with LIF reduces neutrophil recruitment and NETosis, suppressing gastric cancer peritoneal metastasis [143].

NETs also contribute to the awakening of dormant tumor cells. Chemotherapeutic agents such as doxorubicin, cisplatin, and paclitaxel can induce fibroblast senescence, promoting NET formation [144]. These NETs, carrying proteases such as MMP-9, PR3, and NE, remodel the ECM, degrade laminin, and activate α3β1 integrin, directly awakening dormant cells [12]. They may also neutralize microenvironmental inhibitory signals, such as TSP-1, relieving growth arrest and triggering proliferation [102,113].

Nevertheless, these experiments rely on complex in vivo models where NETs are induced alongside other inflammatory mediators, including SASP factors. It remains unclear whether NETs alone are sufficient to trigger dormancy escape. Another unresolved question is whether NETs contribute primarily to early colonization and micrometastasis or also influence later stages of metastatic growth.

## 4. NETs Suppress the Immune System

NETs play a pivotal role in tumor-mediated immunosuppression, contributing to tumor growth, invasion, and metastasis while forming a key component of PMN formation. Through multiple immunoregulatory pathways, NETs inhibit the activity of immune effectors, impair tumor recognition and clearance, and establish an iTGF-β-driven immune-evasive barrier that supports lesion survival and dissemination.

### 4.1. NETs Shield Immune Recognition

NETs act as a physical barrier around tumor cells, preventing direct contact with immune effector cells and thereby suppressing the cytotoxic activity of CD8^+^ T cells and NK cells [53,145]. Chi3l1-induced NETs further impede T-cell infiltration, generating a T-cell-excluded tumor phenotype [102,146]. Strategies that enhance CD8^+^ T cell infiltration or reverse T-cell exhaustion may be crucial for improving responses to immunotherapy [147].

### 4.2. NETs Directly Injure Immune Cells

NET components, including MPO, H3Cit, NE, and TMCO6, interfere with T cell receptor (TCR) signaling, reducing CD8^+^ T cell activity, inhibiting chemotaxis, and inducing apoptosis, which collectively diminish T cell infiltration into tumors [82,148]. Additionally, NETs lower IFN-γ and granzyme B secretion, further compromising cytotoxic function. In NK cells, NETs degrade activating receptors, suppressing cytotoxicity [149]. Tumor-intrinsic overexpression of targets such as Gsk3a, Chi3l1, and CD276 enhances NET formation while reducing NK cell populations, thereby promoting tumor progression [150,151]. Extracellular DNA and granular proteins from NETs drive CD8^+^ T cell exhaustion and can carry PD-L1 to directly inhibit T cells; combined treatment with anti-PD-L1 antibody and DNase I partially restores T cell activity [146,150,151].

### 4.3. NETs Recruit and Promote Immunosuppressive Cell Polarization

NET-derived CXCL12 recruits regulatory T cells (Tregs) and myeloid-derived suppressor cells (MDSCs), further dampening anti-tumor immunity [152]. Chi3l1-induced NETs activate STAT3 signaling and stimulate secretion of additional immunosuppressive factors, reinforcing immune suppression [151,153]. NETs also activate TLR pathways in peritoneal mesothelial cells, inducing CXCL13 secretion that recruits CD43^+^ B cells and promotes IL-10 production, which expands Tregs and suppresses anti-tumor Th1 responses [153]. Furthermore, NET granular proteins, such as calprotectin, directly induce macrophage polarization toward an immunosuppressive M2 phenotype [106].

### 4.4. NET Effects on the Tumor Microenvironment

NET-DNA binding to TMCO6 inhibits CD8^+^ T cell antitumor function, forming a barrier that limits therapeutic efficacy [99]. NETs activate tumor surface receptor CD147, inducing secretion of proinflammatory cytokines IL-6 and IL-8, which stimulate tumor proliferation and enhance tumor-microenvironment interactions, accelerating metastatic lesion formation [138]. HMGB1 released via NETs activates tumor cells through TLR9 signaling, promoting immune evasion and metastasis [154]. In cholangiocarcinoma, CXCL6 autocrine signaling regulates a CXCR1/2-JAK-STAT/PI3K axis that drives lipid metabolic reprogramming and, through RAS/MAPK activation in neutrophils, promotes NET formation; this process subsequently blocks CD8^+^ T cell infiltration and induces resistance to immunotherapy [112,155].

## 5. Current Therapeutic Strategies Targeting NETs

**Figure 4:**
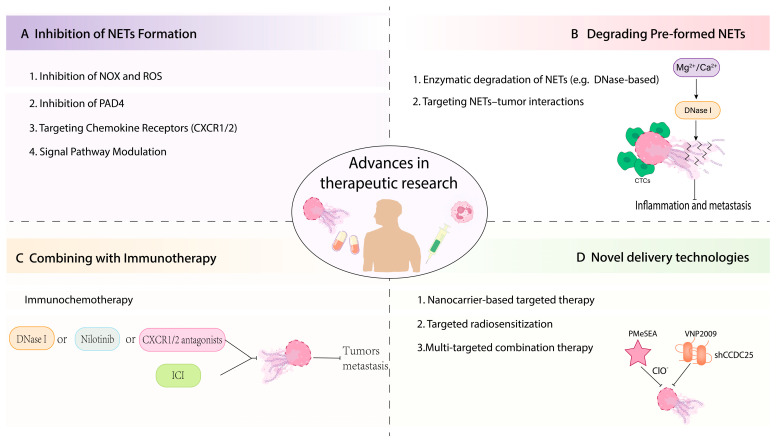
Four complementary strategies to modulate NET formation and clearance and their integration with immunotherapy, aimed at suppressing inflammation and preventing tumour metastasis. (**A**) Reduce NET biogenesis; (**B**) Enzymatically dismantle or degrade pre-formed NETs; (**C**) Combine NET-directed interventions with immune-based therapies to enhance antitumour immunity; (**D**) Employ advanced delivery technologies to improve targeting, efficacy and safety of NET-modulating agents. Arrows indicate: →:Solid arrows; ⊣: Inhibitory symbols.

Pathologically excessive accumulation of NETs, coupled with impaired NET clearance, has been identified as a key mechanism driving malignant cancer progression. This process directly promotes tumor growth and distant metastasis. In addition, by remodeling the tumor microenvironment and inducing an immunosuppressive state, it creates a favorable niche that supports the survival and colonization of micrometastases. Based on this, targeting aberrant NETosis—either by inhibiting its excessive formation or by promoting effective NET clearance—emerges as a highly promising anti-tumor therapeutic strategy. Accordingly, NETosis-related treatment strategies and specific agents (Table 2) can be categorized into four main approaches (Figure 4): (1) reducing NET production; (2) intervening in the NETosis process; (3) dismantling preformed NETs; and (4) combining NET-targeted interventions with other oncologic therapies to improve overall treatment efficacy.

### 5.1. Inhibition of NETosis

Guided by the molecular composition and biogenesis of NETs, strategies that suppress NOX-dependent ROS production and those that block NE- and MPO-mediated chromatin decondensation can effectively limit NET formation, thus potentially reducing the risk of metastasis [85,183].

#### 5.1.1. Targeting NOX and ROS

In classical lytic NETosis, bursts of ROS generated by NOX2 are a principal trigger. Therefore, inhibiting NOX activity or scavenging ROS can interrupt NETosis, representing a potential antimetastatic strategy [131]. Diphenyleneiodonium (DPI), a flavin adenine dinucleotide (FAD) enzyme inhibitor, suppresses NOX-dependent ROS both in vitro and in vivo. When combined with the α5β1 integrin inhibitor ATN-161 (Ac-PHSCN-NH2), DPI has been shown to reduce NET formation and impede colorectal cancer progression in preclinical models [156,184]. However, its clinical utility is limited due to poor specificity, notable off-target effects, and toxicity.

Additionally, other preclinical interventions have demonstrated promising results. For example, exenatide reduced ROS production and NET formation in murine lung (LLC) and colon (MC38) cancer models, and enhanced anti-PD-1 efficacy and CD8^+^ T cell responses when combined with PD-1 blockade [158]. Similarly, DNaseI@Au systems gradually release DNase I, degrade ROS-induced NETs, and block interactions between CTCs and vascular or tissue sites, thereby limiting metastasis in experimental settings [159]. These approaches remain largely preclinical and require further clinical validation.

#### 5.1.2. Inhibition of PAD4-Mediated Histone Citrullination

PAD4-mediated H3Cit is a key driver of chromatin decondensation and NET release. Therefore, inhibiting PAD4 or reducing H3Cit levels can suppress NETosis and potentially decrease metastatic potential. Currently, PAD4 inhibitors are largely confined to in vitro and animal studies. Cl-amidine, an irreversible pan-PAD inhibitor, reduces histone citrullination and NET formation in preclinical models. However, its limited selectivity and unfavorable pharmacokinetic properties raise concerns regarding off-target effects and toxicity [160,161]. GSK484, a more selective and competitive PAD4 inhibitor, has also been reported to decrease NET formation in experimental systems [185]. Both Cl-amidine and GSK484 have been extensively studied in vitro and in vivo, primarily in the context of inflammation-related diseases.

Alternatively, upstream modulation of histone modification pathways can also attenuate NETosis. For instance, icariin, a constituent of Epimedium, binds PADI2 and downregulates its expression, thereby reducing histone citrullination and NET formation [165]. Serotonergic signaling has also been implicated in promoting H3Cit formation, and interventions such as TGM2 knockout or fluoxetine-mediated SERT inhibition decrease these modifications and suppress NET generation in murine models [186].

#### 5.1.3. Targeting Chemokine Receptors That Recruit Neutrophils

The CXCL8-CXCR1/2 axis mediates neutrophil recruitment and NETosis within the tumor microenvironment, thereby promoting metastatic cascades [187]. Preclinical blockade of this axis has been shown to reduce NET-associated metastatic phenotypes. For example, anti-CXCR2 antibodies inhibit IL-8-induced ERK/STAT3 signaling, lower ROS production and NET release, and decrease liver metastases in pancreatic cancer models [167,168]. The CXCR1-biased small molecule inhibitor reparixin markedly reduced neutrophil infiltration (approximately 70%) and lung metastases (>50%) in breast cancer models, although its direct effects on NETosis have not been fully characterized [188].

In addition, epigallocatechin-3-gallate (EGCG), a major active component of green tea, suppressed NETosis in colon cancer-derived neutrophils and reduced STAT3 and CXCL8 expression, thereby inhibiting SW480 cell migration and invasion in vitro [170]. Taken together, these findings support the CXCL8-CXCR1/2 axis as a tractable antimetastatic target. Nevertheless, the tumor-specific, species-relevant, and immunological effects of targeting this axis on NET formation and metastasis require rigorous validation.

#### 5.1.4. Modulation of Upstream Signalling Pathways

Modulating upstream signalling pathways can prevent neutrophils from entering a NET-releasing state, thereby mitigating their pro-metastatic activity. Key pathways implicated in NETosis include NOX2/ROS, PAD4, ERK/MAPK family members, and Ca^2+^ signalling [48,125]. Icariin has been shown to simultaneously attenuate ROS bursts and inhibit MEK/ERK/p38 phosphorylation, reducing NET formation in MB49 bladder cancer cell assays and in C57BL/6J mice [165,171]. Representative constituents of Panax ginseng (ginsenoside Rg1) and Salvia miltiorrhiza (cryptotanshinone, CPT) also suppress metastasis in LCC lung models. Specifically, CPT downregulates the endothelial adhesion molecule CD62E and reduces neutrophil recruitment to the lung, whereas Rg1 inhibits PMA-induced ROS, ERK1/2 and MAPK activation, and decreases chemokines including CXCL1, CXCL2, and G-CSF, collectively limiting NET formation and metastasis [171]. Collectively, targeting upstream signals is mechanistically attractive; however, tumour-contextualized, controlled in vivo studies and thorough safety evaluations are required before clinical translation.

### 5.2. Degradation of Pre-Formed NETs

Degradation of pre-formed NETs represents one of the most common interventions in both basic and animal studies to disrupt the metastatic cascade. By directly dismantling NETs, this strategy not only alleviates the physical trapping of CTCs but also reduces the contribution of NETs to an immunosuppressive and pro-inflammatory microenvironment.

#### 5.2.1. Enzymatic Dismantling of NETs

DNase I enzymatically degrades the DNA backbone of NETs [160], directly disrupting established NETs and reducing their contributions to CTC capture, thrombosis, and pro-metastatic niche formation [133,154]. Its advantages include rapid action and pathway-independent activity, which confer broad potential applicability. However, DNase I-mediated NET degradation may release pro-inflammatory DNA or protein fragments. Recombinant DNase I is further limited by short half-life, proteolytic degradation, potential immunogenicity, and renal clearance [189], motivating the development of systemic or targeted delivery strategies.

To overcome these limitations, two primary approaches have been explored. First, gene-delivery strategies employing adeno-associated virus (AAV) vectors expressing DNase I demonstrated antimetastatic potential in MMTV-PyMT breast cancer models and can be combined with immune checkpoint therapy to synergistically suppress invasion and metastasis [174]. Second, targeted NET-clearance strategies have been proposed. For instance, CT26 cells engineered to overexpress the NET ligand CCDC25 were used to generate membrane vesicles that were co-extruded with DNase I-loaded liposomes, forming CCDC25-DNase I liposomes capable of preferentially targeting and clearing NETs, effectively treating colorectal cancer liver metastases [176]. In another approach, a NIR-II-responsive nanoplatform composed of an AuPB core and an mPDA shell enabled local, light-controlled release of DNase I, disrupting the NET barrier, enhancing immune cell infiltration, and mediating tumour regression and metastasis suppression [178]. Future studies should systematically evaluate safety, immunogenicity, dosing regimens, and efficacy across diverse tumour types to define the clinical potential of these strategies.

#### 5.2.2. Targeting NET-Tumour Interactions

Disrupting NET-tumour interactions represents a critical strategy to prevent metastasis. NET-DNA binding to the tumour cell receptor CCDC25 directly mediates NET-driven pro-metastatic effects, making this axis an attractive therapeutic target. Two main approaches have been reported. The first approach involves palmitic-acid-conjugated mitoxantrone (di-Pal-MTO), which prolongs membrane residence time in multiple mouse models, blocks NET-DNA binding to CCDC25, and inhibits NET-induced RAC1-CDC42 signalling, cytoskeletal rearrangement, and chemotactic migration. By modulating inflammatory cues, this approach also enhances dendritic cell activation and CD8^+^ T cell infiltration [80]. However, the compound and/or its metabolites may exhibit cytotoxicity, warranting further evaluation of safety and selectivity. The second strategy employs the oncolytic/attenuated bacterial vector VNP20009 to deliver shRNA against CCDC25 (VNP-shCCDC25) in murine tumour models. Local knockdown of CCDC25 inhibits downstream pro-metastatic signalling and reshapes the immune microenvironment, increasing intratumoural neutrophil and macrophage infiltration while reducing NET deposition. This dual effect produces both antimetastatic and immune-activating outcomes [180]. While these strategies highlight the potential of targeting NET-DNA-CCDC25 interactions, their generalizability across tumour types and long-term safety remain to be established.

### 5.3. Combination with Immunotherapy

Targeting NET-tumour interactions can remodel the immune microenvironment and increase immune cell infiltration, providing a rationale for combining NET-directed interventions with immune checkpoint inhibitors (ICIs) to suppress metastasis [180]. Strategies to enhance immunotherapy through NET targeting can be classified into four complementary categories.

(1) Direct degradation or blockade of NET components and signalling. For example, DNase I degrades NETs and can interrupt the NETs-PD-L1 axis, restoring T cell function and relieving NET-driven immunosuppression. (2) Inhibition of cell recruitment or receptor pathways that promote NET formation. In a phase I/II trial of patients with advanced cancer, the CXCR1/CXCR2 inhibitor reparixin combined with anti–PD-1 therapy reduced peripheral blood levels of the NET marker CitH3 and enhanced CD8^+^ T cell-mediated antitumor activity [53]. Similarly, the fully human anti-IL-8 monoclonal antibody HuMax-IL8 (BMS-986253, NCT02536469) neutralizes IL-8, inhibiting neutrophil recruitment and activation. Phase I data indicated excellent safety, decreased serum IL-8, and reversal of IL-8/NET-mediated immune suppression, creating a favourable microenvironment for T cell activation. While objective responses were not observed with monotherapy, 73% of patients achieved disease stability, with a 5.5-month progression-free survival of 53.3%, and no maximum tolerated dose was reached [190]. No real-world data are yet available.

(3) Targeting tumour stroma or signalling to indirectly suppress NETosis. For instance, DDR1 inhibition by nilotinib combined with ICIs reversed a liver fibrosis-associated, neutrophil/NET-dominant immunosuppressive microenvironment and markedly improved anti-PD-1 responses in preclinical HCC models [150].(4) Small molecules or natural products with synergistic activity. Icariin reduced neutrophil infiltration and NET formation in animal models, synergizing with anti-PD-1 to relieve T cell suppression and inhibit tumour growth [165].

Collectively, these approaches—whether by degrading NETs, blocking NET-associated pathways, or reducing neutrophil recruitment—show considerable potential to enhance ICI efficacy. Future work should focus on identifying predictive biomarkers (e.g., CitH3) for patient stratification, optimizing timing and dosing in combination regimens, and validating clinical benefit in randomized controlled trials.

### 5.4. Novel Delivery Technologies

#### 5.4.1. Nanocarrier-Based Targeted Therapies

Advanced nanocarrier systems provide a promising approach to selectively suppress NETs and inhibit metastasis. For example, the sulfoxide-containing polymer PMeSEA exhibits potent NET-inhibitory activity and excellent anti-biofouling properties. MPO generates HOCl from H_2_O_2_ and Cl^−^, and HOCl contributes to lytic NETosis. The hydrated sulfoxide groups in PMeSEA reduce nonspecific protein and cell adhesion while scavenging HOCl species required for NET stability, thereby blocking NETosis without perturbing cellular redox homeostasis. In preclinical models, PMeSEA markedly reduced peritoneal metastasis [181]. These properties underscore the potential of nanocarrier-based strategies for targeted NET modulation.

#### 5.4.2. Enhancing Radiotherapy by Targeting NETs

Radiotherapy effectively eliminates local tumour cells but can paradoxically induce NET formation via ROS generation. Residual NETs, together with elevated cancer-associated neurotransmitters, may activate β-adrenergic receptors, recruit pro-tumour MDSCs, and promote angiogenesis, facilitating distant metastasis [176]. To counteract this, aerosolized DNase I-loaded Au nanoparticles (DNaseI@Au) have been delivered to the lung during radiotherapy in preclinical models. This approach effectively degraded NETs, disrupted interactions between free tumour cells and the vasculature, reduced metastatic risk, and enhanced radiotherapy efficacy, resulting in significant tumour shrinkage [159]. These findings support further development and clinical translation of NET-targeted adjuncts for radiotherapy.

#### 5.4.3. Multi-Target Combined Strategies

Given the multifactorial nature of metastasis, single-target interventions are often insufficient. Multi-target platforms have been engineered to simultaneously disrupt complementary pro-metastatic mechanisms. For instance, an injectable interpenetrating network (IPN) hydrogel was designed for local co-delivery of DNase I and a β-adrenergic receptor antagonist (PR) in a 4T1 resection model. The hydrogel formed an adhesive matrix that sustained release of DNase I and PR, degraded NET-DNA, antagonized cancer-associated neurotransmitters, reduced microvessel density and angiogenesis, lowered MDSC levels and pro-inflammatory cytokines, enhanced NK cell function, mitigated T cell exhaustion, reprogrammed the tumour immune microenvironment, suppressed local residual disease and distant metastasis, and induced immune memory to decrease recurrence [180].

Another dual-delivery system, 5HT-NP@D+p-TC-RLA, simultaneously targeted NETs and tumour mitochondria. 5HT-NP@D released DNase I specifically at NET sites, while p-TC-RLA delivered a mitochondria-targeted peptide (RLA) to inhibit oxidative phosphorylation and relieve hypoxia. This approach disrupted the NET-hypoxia-mitochondrial metabolism positive feedback loop, markedly suppressing tumour migration, invasion, and distant metastasis in an orthotopic breast cancer model [191]. While these multi-target platforms show strong preclinical potential, their clinical translation requires comprehensive safety, pharmacokinetic, and manufacturing evaluations.

### 5.5. Summary and Future Directions

Targeting NETs represents a promising strategy to inhibit tumour metastasis. Preclinical studies indicate that suppression of NOX-dependent ROS, PAD4-mediated histone citrullination, neutrophil chemotaxis, and upstream ERK/MAPK or Ca^2+^ signalling can reduce NETosis and metastatic potential. Interventions include small molecules such as DPI, Cl-amidine, GSK484, and natural compounds like icariin. Degradation of established NETs through DNase I or targeted delivery systems—including lipid nanoparticles, adeno-associated virus vectors, or NIR-II-responsive nanoplatforms—disrupts circulating tumour cell capture and pro-metastatic niches, although systemic administration is limited by short half-life and potential immunogenicity.

Blocking NET-tumour interactions with agents such as di-Pal-MTO or VNP-shCCDC25 inhibits pro-metastatic signalling and remodels the immune microenvironment, yet their safety and long-term efficacy require further validation. Clinically, CXCR1/2 inhibitors (e.g., reparixin, NCT02370238) and IL-8 neutralizing antibodies (HuMax-IL8) have demonstrated favorable safety profiles and immunomodulatory effects in early-phase trials. Combining NET-targeted strategies with ICIs, alongside advanced delivery platforms such as nanocarriers or hydrogels, may enhance therapeutic efficacy, overcome pharmacokinetic limitations, and potentiate anti-tumor immunity. Integrating mechanistic insights, biomarker-guided patient stratification, and controllable delivery systems constitutes a critical path toward the clinical translation of NET-targeted therapies.

## 6. Conclusions and Future Directions

Recent evidence highlights the dual role of NETs in cancer, modulating both tumor progression and metastasis. While NETs predominantly promote tumor growth in early-stage and inflammation-associated cancers, their potential antitumor functions remain underexplored [192]. NET formation is orchestrated by key pathways, including the CXCL8-CXCR1/2 axis and PAD4-H3Cit pathway. Elevated CXCL8 correlates with increased NET burden, presenting a potential therapeutic target, whereas H3Cit may serve as a predictive biomarker for metastatic disease.

NET function is context-dependent, influenced by tumor type, stage, TAN phenotype, NET formation mechanisms, and the inflammatory status of the TME. In pancreatic and breast cancers, NETs mainly enhance tumor progression, whereas in melanoma, antitumor effects are more pronounced. N1-TAN-derived NETs exert antitumor properties, while N2-TANs facilitate tumor growth. NET morphology and abundance further modulate outcomes: excessive NETs accelerate progression, whereas moderate levels contribute to immune regulation. TME inflammation additionally shapes NET function, with chronic inflammation amplifying pro-tumor effects and moderate inflammation enhancing antitumor immunity.

Critically, NET biology is highly environment-dependent, varying with tumor type, metastatic stage, and systemic inflammation [27,34]. For instance, NETs drive metastasis in inflammation-driven tumors, such as hepatocellular carcinoma with chronic hepatitis, but their roles in low-inflammatory tumors or late-stage colonization remain unclear. This underscores the need to dissect bidirectional interactions between NETs and the TME and to define the contributions of individual NET components to tumor cell behavior, which is essential for rational therapeutic design.

Preclinical studies demonstrate that targeting NOX-dependent ROS production, PAD4-mediated histone citrullination, neutrophil recruitment, upstream ERK/MAPK or Ca^2+^ signaling, or degrading pre-formed NETs via DNase I and advanced delivery systems can effectively suppress metastasis. Early clinical trials indicate that CXCR1/2 inhibitors and IL-8 neutralizing antibodies are safe and immunomodulatory, and their combination with immune checkpoint inhibitors or multi-targeted strategies may further enhance antitumor efficacy.

Nonetheless, the majority of evidence remains preclinical, and the safety, specificity, and long-term efficacy of NET-targeted interventions require systematic evaluation. Future studies should investigate NET heterogeneity, clarify the balance between pro- and anti-tumor functions, and evaluate translational potential, thereby informing the development of effective, environment-specific NET-targeted therapies.

## Figures and Tables

**Table 1:** Factors stimulating NETs and their mechanisms of action.

Type	Source Factor	Specific Factor	Mechanism of Action	Result	References
Non-tumor cell derived factors	Chronic psychological stress & inflammatory cytokines	IL-6, IL-1β, TNF-α	ROS, activate p38 MAPK and cyclin D3/CDK4/6	NETosis	[92,93,94]
	Gut dysbiosis	——	Neutrophil inflammatory and Ly6G^+^	Increased NET formation	[95]
	Activated platelets	HMGB1, P-selectin	Released mediators	Establish a sustained NETosis-promoting loop	[96,97]
	Chemotherapy	ATP	(1) ATP activates NLRP3 and IL-1β; (2) IL-1β recruits neutrophils	Induction of NETosis	[98,99]
Tumor cell-derived factors	Tumor cells	G-CSF, CXCL1	Neutrophil production	Increase supply of neutrophils for NETosis	[100]
	Tumor cells	CTSC	Activates neutrophil PR3 and NF-κB, upregulates IL-6 and CCL3	Amplifies inflammation and NETosis	[82,101,102]
	Tumor cells (via TRAPs)	HMGB1	ROS and TLR4/MyD88	Enhanced inflammation and NETosis	[103,104]
	Tumor cells	NQO1	PPIA activates neutrophil CD147	NET induction and protease release	[105]
	Tumor cells	FGF19	Activates FGFR4-JAK2-STAT3	converts them to inflammatory CAFs	[106]
	iCAFs (induced by FGF19)	C5a, IL-1β	——	Stimulate NETosis and MPO release	[107]
	Liver-metastatic breast cancer cells	DMBT1	DMBT1 binds MUC1 on Kupffer cells, activates NF-κB and CCL8	Via CCL8-CCR1-ERK axis induces NETosis	[31,108]
NETs/TME	NETs	HMGB1	Activates TLR9 and IL-8	Positive feedback loop expanding NET accumulation	[109,110]
	NETs	NE	Activates TLR4 and IL-8/CXCL6	Positive feedback, expanding NET accumulation	[111,112]

iCAFs, Inflammatory cancer-associated fibroblasts; TRAPs, Tumor cell-released autophagosomes; MyD88, Myeloid differentiation primary response 88; CDK, Cyclin-dependent kinase; NLRP3, NOD-like receptor pyrin domain-containing 3; CTSC, Cathepsin C; NQO1, NAD(P)H quinone dehydrogenase 1; PPIA, peptidyl-prolyl cis-trans isomerase A.

**Table 2:** Drugs Targeting NET-Mediated Tumour Metastasis.

Type	Intervention	Mechanism	Result	Applications	References
Inhibition of NOX and ROS	DPI	NOX, ROS	Inhibition of NETs formation	colorectal and breast cancer metastasis	[156,157]
	Exenatide, DNaseI@Au	ROS	Inhibition of NETs formation	LLC (lung) and MC38 (colon) cells; lung cancer	[158,159]
Inhibition of PAD4	Cl-amidine	PAD-mediated H3Cit	Prevents chromatin decondensation and NET release	Ovarian peritoneal dissemination; ESCC; HCC; colorectal cancer	[160,161,162]
	GSK484	PAD4-driven H3Cit	Prevents chromatin decondensation and NET release	ESCC; HCC	[146,163,164]
	Icariin	PADI2, H3Cit	Inhibition of NETs formation	HCC	[165]
	Fluoxetine (SERT inhibitor)	5-HT axis	Modulates histone marks; inhibits NETs	Thyroid medullary carcinoma liver metastasis	[166]
Targeting Chemokine Receptors	Anti-CXCR2 antibody	CXCR2/IL-8; ROS	Reduces inflammation, NET release, and metastasis	HCC lung metastasis; PDAC	[167,168]
	Reparixin	CXCR1/2 reduces neutrophils	Inhibits NETosis	Septic thrombosis	[169]
	EGCG	STAT3-CXCL8	Impedes NET formation and cell migration	Colon cancer migration and invasion	[170]
Signaling Pathway Modulation	Icaritin	ROS, MAPK	Blocks NET-induced metastasis	Urothelial carcinoma	[165]
	Ginsenoside Rg1	ROS, MEK/ERK/p38	Inhibits NETosis initiation	Lung cancer metastasis (in vivo)	[171]
	Baicalin	IFN-I-JAK-STAT, ERK/PI3K	Reduces NET formation	Anti-inflammatory, immunomodulatory contexts	[172]
	DNase I+ anti-PD-1	CD73 activate NF-κB pathway	inhibit NETs and improves anti-PD-1 therapy efficacy	HCC immunotherapy study	[173]
	AAV-DNase I	Gene delivery (with ICI)	Sustained DNase I expression; potential long-term metastasis suppression	Colorectal cancer liver metastasis	[174,175]
	CCDC25-DNase I Liposome	Targeted delivery	Targeted clearance of NETs	Colorectal cancer liver metastasis	[176,177]
	AuPB@mPDA vector	NIR-II-triggered DNase I release	On-demand NET barrier disruption	Colorectal cancer	[178]
Targeting NET-Tumor Interactions	di-Pal-MTO	NET-DNA inhibits RAC1-CDC42	Reduces chemotaxis; promotes DC activation and CD8^+^ T infiltration	Breast cancer metastasis	[80]
	NET blockade	Inhibite CD4^+^ T cells and Treg differentiation	Reduced Treg activity	NASH-HCC	[179]
	VNP20009 delivering shCCDC25	CCDC25 knockdown	Blocks NET-driven metastasis and remodels TME	Pulmonary metastasis	[180]
Nanocarrier Targeted Therapy	PMeSEA Polymer	ClO^−^ preserves redox balance	Inhibits NET formation; reduces peritoneal metastasis	Peritoneal metastasis	[181]
	DM/HA-PLIP Nano Delivery System	ECM and angiogenesis, α-SMA	Reduces metastatic nodules and NET burden	Breast metastasis	[182]
Targeted Radiotherapy Enhancement	Inhaled DNase I@Au Nanoparticles	Targeted lung tumors; clears NETs	Disrupts tumor-vascular interactions; lowers metastasis risk and enhances RT efficacy	Lung cancer metastasis	[159]
Multi-target Combination Therapy	IPN Hydrogel (DNase I + PR)	Disrupts NETs and antagonizes CANTs	Inhibits residual and distant metastasis; induces immune memory	Postoperative recurrence and metastasis	[180]
	5HT-NP@D+p-TC-RLA	DNase I release, reduces O_2_ consumption	Degrades NETs and breaks the metastasis-promoting cycle	Prostate cancer liver metastasis	[166]

PADI2, Peptidyl Arginine Deiminase Type 2; DNaseI@Au, DNase I-loaded gold nanoparticles; (ICIs), immune checkpoint inhibitors; CANTs, cancer-associatd neurotransmitters.

## Data Availability

The data used in this review are derived from public resources, including but not limited to well-known academic databases, publicly available government reports, and authoritative websites in specialized fields. The acquisition of all data complies with the relevant laws and regulations, as well as the terms of use stipulated by the data providers. For the data obtained from academic databases, their sources have been detailed in the references, and readers can trace the original data based on the cited information. Data from government reports can be accessed through the official websites of the respective government departments. Data from authoritative websites in specialized fields are provided with links or clear source descriptions at appropriate locations in the text. The purpose of this statement is to ensure the transparency and reproducibility of the data, thereby promoting the healthy development of academic research. If you have any special requirements regarding the sources and acquisition methods of the data in this report, or if you would like to provide additional information, please feel free to let me know.
